# Dystonia as an early and prominent feature in a patient with CYP2U1 gene mutation: expanding the phenotype of SPG56-a case report

**DOI:** 10.1186/s13023-026-04349-8

**Published:** 2026-04-27

**Authors:** Zeina Alhamwy, Alaa Senjab, Ahmad Al-Bitar, Diana Alasmar

**Affiliations:** Faculty of Medicine, Damascus University, Damascus, Syrian Arab Republic

**Keywords:** Dystonia, Genetic disease, Genetics, HSP, CYP2U1, Neurology, Mutations, Case report

## Abstract

Hereditary Spastic Paraplegias (HSPs) are a heterogeneous group of neurodegenerative disorders primarily characterized by progressive lower limb spasticity and weakness. Among them, Spastic Paraplegia Type 56 (SPG56) is a rare autosomal recessive form caused by CYP2U1 mutations. While spasticity is the hallmark of SPG56, recent reports have shown expanding phenotypic variability, including dystonia. We report an 8-month-old Arab male who presented with generalized tonic seizures and abnormal head movements, later identified as dystonic in nature. MRI revealed cerebral atrophy and delayed myelination. Given the early symptom onset and unexplained neurodevelopmental presentation, exome-based next-generation sequencing was performed. This identified a homozygous missense variant in CYP2U1 (c.947 A > T; p.Asp316Val), classified as likely pathogenic. This variant has previously been associated with SPG56 but rarely with dystonia as a predominant feature. Over 6 months of follow-up, seizure frequency decreased markedly and dystonia improved substantially on sodium valproate and levetiracetam, although mild dystonic movements persisted and no lower-limb spasticity was observed. This case highlights the diagnostic value of exome-based genetic testing in unexplained early-onset neurodevelopmental disorders. It also underscores the phenotypic heterogeneity of SPG56 and suggests that dystonia may, in some cases, be an early and dominant manifestation of CYP2U1-related pathology. Reporting such rare presentations is essential to broaden our understanding of the clinical spectrum of CYP2U1 mutations.

## Introduction

Hereditary Spastic Paraplegias (HSPs) encompass a clinically and genetically heterogeneous group of neurodegenerative disorders primarily characterized by progressive bilateral lower limb spasticity and weakness, resulting from the degeneration of corticospinal tract axons [1, 2]. With over 80 identified genetic loci (SPG1-SPG80+), HSPs can be inherited in autosomal dominant, autosomal recessive, or X-linked manners and are classified as “pure” when neurological impairment is largely restricted to the pyramidal tracts, or “complicated” when accompanied by additional neurological or systemic features such as intellectual disability, ataxia, seizures, peripheral neuropathy, or white matter abnormalities [2, 3].

Spastic Paraplegia type 56 (SPG56) is a rare, autosomal recessive complicated form of HSP caused by biallelic mutations in the *CYP2U1* gene [4, 5]. The *CYP2U1* gene encodes a cytochrome P450 enzyme, the only known member of its subfamily, which is thought to play a role in fatty acid metabolism and neurodevelopment [6]. Clinically, SPG56 typically presents in early childhood with progressive spastic paraparesis, often accompanied by intellectual disability, developmental delay, cerebellar ataxia, and neuroimaging findings such as thin corpus callosum, periventricular white matter changes, or delayed myelination [5, 7].

While progressive spasticity is the hallmark, the phenotypic spectrum associated with *CYP2U1* mutations is expanding. Dystonia, a movement disorder characterized by sustained or intermittent muscle contractions causing abnormal movements or postures, has been reported as an associated feature in some individuals with SPG56, but it is less commonly the predominant or initial presenting symptom [7, 8]. Early-onset presentations where dystonia is a prominent feature, particularly in infancy, contribute significantly to understanding the full clinical breadth of *CYP2U1*-related neurodegenerative disorders.

Here, we present the case of an 8-month-old male child who presented with early-onset generalized tonic seizures and prominent dystonia, subsequently found to have a homozygous likely pathogenic variant in the *CYP2U1* gene (c.947 A > T, p.Asp316Val).

## Case presentation

**Fig. 1:**
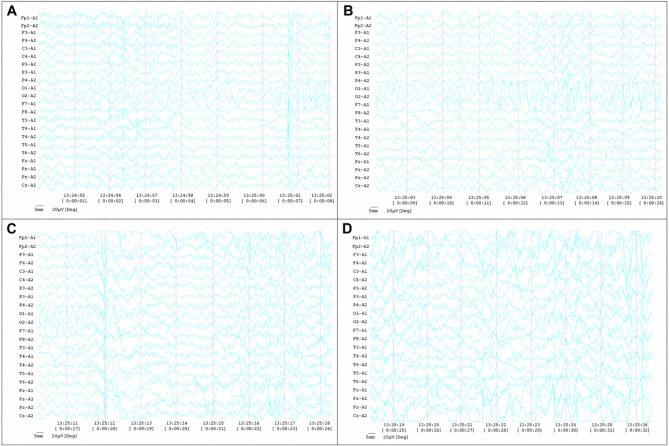
EEG patterns showing abnormal electrical activity. These images present an EEG recording from the patient. The traces show irregular brain waveforms, including sharp waves and spikes in multiple regions, such as Fp1-A1, F3-A1, and Cz-A2. These abnormalities are indicative of epileptiform activity or potential seizure activity. The findings suggest possible neurological dysfunction, such as epilepsy or other related disorders, which require further clinical investigation and diagnosis

An 8-month-old Arab male child presented to the pediatric clinic with generalized tonic seizures and abnormal head movements that started a month ago. The examination showed regression of psychomotor development and cyanosis, and confirmed that the abnormal head movements were typical of dystonia. The patient was put on sodium valproate and levetiracetam. The MRI findings suggested cerebral atrophy and delayed myelination, with no evidence of space-occupying lesions, hydrocephalus or structural brain abnormalities. The patient had a family history of a brother with cerebral atrophy, inability to walk, intellectual disability, loss of speech and seizures. His other two siblings are totally healthy. His parents were not relatives and there was no family history of genetic diseases. He was then referred to a metabolic diseases doctor. Blood tests showed abnormal leukocyte count, abnormal platelet count, decreased mean corpuscular hemoglobin concentration, decreased mean platelet volume, hyperoxemia, hypouricemia and neutrophilia. Amino acid tests came up normal. Other remarkable test results are showed down in Tables 1 and 2. The results of the Tandem Mass Spectrometry (MS/MS) were not remarkable. The patient’s EEG recordings (Fig. 1) showed generalized abnormal brain activity. The traces from the electroencephalogram indicated irregular, high-amplitude slow waves and sharp wave discharges, particularly noted in the frontal and central leads (e.g., FP1-A1, FP2-A1, C3-A2). These findings are consistent with generalized cortical dysfunction, which were associated with the patient’s clinical presentation of seizures and dystonic movements. A blood sample was sent to a genetic laboratory. No clinically relevant variant explaining the phenotype was identified, therefore exome sequencing was recommended. Genetic testing was performed using the CentoXome^®^ MOx 1.0 Solo assay (CentoGene), an exome-based next-generation sequencing test covering approximately 41 Mb of coding exons (RefSeq) and the mitochondrial genome. Sequencing was conducted on an Illumina platform using a peripheral blood sample, with > 98% of targeted bases covered at ≥ 20×; in our patient, 99.38% of targeted nucleotides achieved ≥ 20× coverage. Variant interpretation and classification were performed according to ACMG/AMP criteria. Genetic testing (Table 3) revealed a homozygous likely pathogenic variant in the CYP2U1 gene: c.947 A > T, resulting in the amino acid substitution p.Asp316Val. This variant is classified as Class 2 (likely pathogenic) based on ACMG guidelines. Supporting evidence includes the absence of this variant from population databases (gnomAD, ESP, 1000 Genomes, and CentoMD) and concordant deleterious predictions across multiple in silico tools (SIFT, PolyPhen-2, and MutationTaster), with high evolutionary conservation (Table 3).

It has been previously associated with autosomal recessive spastic paraplegia type 56 (SPG56), a neurodegenerative disorder characterized by progressive lower limb spasticity, motor delay, and variable neurological manifestations. Although no other clinically relevant variants were identified to fully explain the patient’s phenotype, the presence of this CYP2U1 variant suggests a possible contributory role and warrants further clinical correlation, including assessment for phenotypic overlap and targeted parental testing to confirm carrier status and homozygosity.

Over 6 months of follow-up, the patient remained on sodium valproate and levetiracetam without treatment modification. Seizure frequency decreased markedly, and dystonia improved substantially, although mild dystonic movements persisted. No new neurological symptoms were reported, and no clear lower-limb spasticity was observed. Overall, the course was stable with partial symptomatic improvement.

**Table 1:** Abnormal & clinically relevant results

Test	Result	Reference Range	Unit	Interpretation / Relevance
CK (Total)	1964	25–195	U/L	Very high → Suggests muscle breakdown or metabolic myopathy
Ammonia	81.10	30–70	mcg/dL	High → Possible urea cycle disorder or liver/metabolic dysfunction
Iron (Fe)	45	65–165	µg/dL	Low → May contribute to developmental or neurological symptoms
Chloride (Cl⁻)	97	98–106	mmol/L	Low → Mild hypochloremia, often non-specific
pH (arterial)	7.5	7.35–7.45	—	High → Respiratory alkalosis
pCO₂	29.3	35–45	mmHg	Low → Consistent with respiratory alkalosis
HCO₃⁻	20.3	22–26	mmol/L	Low → May reflect compensation for respiratory alkalosis
Urine Citric Acid	Elevated	—	—	Non-specific, may suggest mitochondrial dysfunction

**Table 2:** Tests that should be abnormal (but were normal or not done)

Test	Result/Status	Reference Range	Comment / Why Relevant
Lactic Acid	22.6	5.4–28.8 mg/dL	Normal, but often elevated in mitochondrial disorders
Tandem MS / Newborn Screening	Not Remarkable	—	Many aminoacidopathies & organic acidemias ruled out
Urine Organic Acids	Mostly normal	—	Citric acid elevated; others normal, which does not rule out all metabolic disorders

**Table 3:** Genetic test results

Sequence variants								
Gene	Variant Coordinates	Amino Acid Change	Zygosity	In Silico Parameters*	Allele Frequences**	Type And Classifi cation***	Related Disorder (OMIM^®^) And Mode Of Inheritance	
CYP2U1	NM_183075.2:c.947 A> T^1^	p.Asp316Val	Homozygous	PolyPhen: Probably damaging Align-GVDG: N/A SIFT: Deleterious MutationTaster: Disease causing Conservation_nt: high Conservation_aa:	gnomAD : - ESP: - 1000 G: - CentoM D: -	Missense Likely Pathogenic (class 2)	Spastic paraplegia 56 (615030) AR	

## Discussion

Dystonia is a movement disorder characterized by sustained or intermittent muscle contractions causing abnormal, often repetitive movements or postures [9, 10]. It may occur as an isolated condition or as part of a broader neurogenetic syndrome [10]. In hereditary spastic paraplegias (HSPs), particularly SPG56 caused by *CYP2U1* mutations, dystonia is a less commonly reported but clinically significant feature. CYP2U1 is thought to participate in the metabolism of long-chain fatty acids. Disruption in this pathway could impact neuronal membrane integrity, particularly in basal ganglia circuits, which may explain the prominent dystonic phenotype observed in our patient [11, 12]. Dystonia has been reported as a prominent presenting feature of *CYP2U1* mutations in quite few previous case reports [4, 13] (Table 4).

In the first, Kariminejad et al. described two unrelated Iranian children with SPG56 who exhibited normal development until approximately 11 months of age, followed by progressive motor regression, activity-induced limb dystonia, spastic paraplegia, and cognitive impairment [4]. In contrast, our patient demonstrated a significantly earlier onset of symptoms at 7 months, indicating a potentially more severe and rapidly progressive disease course. Genetic analysis in Kariminejad’s et al. report identified novel, likely loss-of-function variants, whereas our case involves a missense mutation classified as likely pathogenic, suggesting a different molecular mechanism of dysfunction. Although neuroimaging was not fully detailed in their report, both children showed subtle white matter abnormalities consistent with delayed myelination—a feature also observed in our patient, but more clearly documented by MRI.

The second report, by Uchida et al., described a 44-year-old male of Chinese descent with adult-onset SPG56, who presented at age 40 with progressive right-hand dystonia resembling writer’s cramp, as well as mild dysarthria [13]. This presentation contrasts sharply with our case, which involved early-onset, generalized dystonia in infancy. Unlike the pediatric onset and severe regression seen in our patient, Uchida’s case had a more focal and isolated presentation. Genetic testing identified compound heterozygous *CYP2U1* mutations (a missense and a novel nonsense variant), both predicted to result in loss of function. Neuroimaging showed basal ganglia calcifications and mild cerebellar atrophy—findings not present in our patient, who instead showed delayed myelination without calcifications or atrophy. Furthermore, the adult patient responded well to deep brain stimulation (DBS), with significant improvement in dystonia. Such intervention is not applicable in our case given the early age, diffuse involvement, and expected disease progression. Phenotypic expression in CYP2U1-related disorders may be highly variable, and severity or age at onset may not necessarily correlate in a straightforward manner with the predicted functional consequence of the underlying variants. Accordingly, the milder adult-onset dystonia described by Uchida et al., despite loss-of-function variants, could plausibly reflect variable expressivity influenced by genetic background (modifier variants), partial residual CYP2U1 activity or compensatory pathways, and/or differences in the timing of clinical ascertainment and reporting. Further genotype-specific functional work and additional well-phenotyped cases would be required to clarify these possibilities.

In contrast to both of these dystonia-predominant presentations, Minase et al. described a Japanese child with SPG56 who presented primarily with severe developmental delay and spastic quadriparesis, rather than dystonia [14]. MRI revealed delayed but non-progressive cerebral myelination and no corpus callosum thinning—a rare neuroimaging finding for SPG56, but similar to the delayed myelination pattern observed in our patient. This further supports the phenotypic variability of *CYP2U1*-related disorders. Like our case, the child reported by Minase et al. also harbored homozygous mutations, reinforcing the association between *CYP2U1* mutations and early-onset neurodevelopmental impairment.

**Table 4:** Comparison of the present case with selected previously reported SPG56/CYP2U1 cases

Study (First author, Year)	*N*	Country/ethnicity	CYP2U1 genotype	Age at onset	Key clinical features	Dystonia	Key MRI findings	Treatment/outcome
Present case	1	Arab male	Homozygous c.947 A > T (p.Asp316Val)	7 mo	Generalized tonic seizures; prominent dystonic movements (abnormal head movements); spasticity not yet documented	Generalized; early and prominent	Cerebral atrophy; delayed myelination; no hydrocephalus/space-occupying lesion	Valproate + levetiracetam; follow-up ongoing
Kariminejad, 2016	2	Iranian children	Biallelic variants (likely loss-of-function)	~ 11 mo	Motor regression; spastic paraplegia; cognitive impairment; activity-induced dystonia	Activity-induced (limb/neck)	Subtle white matter abnormalities consistent with delayed myelination	Progressive course; supportive care (NR)
Uchida, 2024	1	Chinese male	Compound heterozygous (missense + nonsense; predicted loss-of-function)	40 y	Adult-onset focal right-hand dystonia (writer’s cramp–like); mild dysarthria	Focal; task-related	Basal ganglia calcifications; mild cerebellar atrophy	DBS with significant improvement
Minase, 2017	1	Japanese child	Biallelic variant(s) (reported as homozygous)	NR	Severe developmental delay; spastic quadriparesis; dystonia not predominant	Not predominant/absent	Delayed but non-progressive cerebral myelination; no corpus callosum thinning	NR

Abbreviations: NR, not reported or not emphasized in the cited report; DBS, deep brain stimulation

From a genotype–phenotype perspective, the CYP2U1 c.947 A > T (p.Asp316Val) variant has been previously described in the homozygous state in affected families and maps to the cytochrome P450 functional domain, supporting a biologically meaningful impact on protein function [12]. However, SPG56 is characterized by substantial intra- and inter-familial variability, and dystonia may be absent even among affected siblings carrying an identical CYP2U1 genotype, consistent with variable expressivity and the possible influence of additional modifiers [4]. Therefore, we consider the early, dystonia-dominant presentation in our patient to represent an extreme within the expanding SPG56 spectrum rather than a definitive phenotype uniquely attributable to p.Asp316Val, pending further genotype-specific functional and clinical correlation.

Our case differs notably in several aspects: earlier onset (7 months vs. 11 months or later), a missense mutation rather than a truncating or nonsense variant, and clearly documented delayed myelination on MRI. Based on the available literature and the classification of the mutation as likely pathogenic, we anticipate progressive neurological deterioration in our patient, similar to the severe phenotypes observed in pediatric-onset cases. Continued follow-up will be critical to monitor motor and cognitive decline and to explore potential supportive or symptomatic interventions.

## Limitations

Ongoing follow-up should include periodic neurological evaluation, developmental assessment, and MRI monitoring to assess disease progression. While current therapeutic options remain supportive, future developments in gene-targeted therapies may offer disease-modifying potential.

“This report has several limitations. First, although 6 months of follow-up showed a stable clinical course with partial symptomatic improvement on sodium valproate and levetiracetam, longer-term follow-up is still needed to determine the future evolution of the disease and whether more typical SPG56 features, particularly lower-limb spasticity, may emerge over time. Second, no updated longitudinal or molecular data were available for the similarly affected brother at the time of manuscript revision, which limited intrafamilial genotype–phenotype comparison. Finally, although the MRI findings were documented in the radiology report and incorporated into the case description, the original MRI images could not be retrieved for figure inclusion because they remained with the family and were subsequently misplaced.

## Conclusion

In conclusion, the early onset of symptoms and the rapid deterioration of multiple functions in the patient pointed toward an underlying genetic etiology. This underscores the essential role of genome sequencing in establishing a definitive diagnosis by identifying pathogenic mutations. Although most documented cases of SPG56 and CYP2U1 mutations have been primarily associated with spastic paraplegia, our patient presented predominantly with dystonia. This highlights the broad phenotypic variability associated with different CYP2U1 variants. Given the rarity of such genotype-phenotype correlations, reporting and documenting similar cases is crucial to enhance our understanding of the clinical spectrum of CYP2U1-related disorders. This case highlights the importance of considering CYP2U1 mutations in the differential diagnosis of infantile dystonia, particularly when standard metabolic evaluations are inconclusive, and MRI findings suggest delayed myelination.

## Data Availability

Not applicable.

## Abbreviations

HSP: Hereditary Spastic Paraplegia
MRI: Magnetic resonance imaging
EEG: Electroencephalogram
